# Association between chronic diseases and lifestyle risk factors among community-dwelling older adults: a retrospective cross-sectional Chinese population-based study

**DOI:** 10.3389/fpubh.2025.1435385

**Published:** 2025-03-05

**Authors:** Wei Xin, Dan Xu, Zulin Dou, Angela Jacques, Josephine Umbella, Yuling Fan, Longsheng Zhang, Haiwen Yang, Hong Cai, Anne-Marie Hill

**Affiliations:** ^1^Curtin Medical School, Curtin University, Perth, WA, Australia; ^2^Rehabilitation Medicine Department, The Third Affiliated Hospital of Sun Yat-Sen University, Guangzhou, China; ^3^School of Allied Health, The University of Western Australia, Perth, WA, Australia; ^4^Curtin School of Population Health, Faculty of Health Sciences, Curtin University, Perth, WA, Australia; ^5^Medical Education and General Practice Department, The First Affiliated Hospital of Sun Yat-Sen University, Guangzhou, China; ^6^Institute for Health Research, The University of Notre Dame Australia, Fremantle, WA, Australia; ^7^Medical Department, Guangzhou Tianhe District Shipai Street Community Health Service Center, Guangzhou, China; ^8^Medical Department, Guangzhou Tianhe District Linhe Street Community Health Service Center, Guangzhou, China

**Keywords:** aged, public health, chronic disease, risk factors, community

## Abstract

**Background:**

Chronic diseases among older adults are recognised as a serious public health problem in China, causing rising social and economic burden. The aim of the study was to evaluate the association between chronic diseases and lifestyle risk factors among community-dwelling older adults who attended community health service centres (CHSC) in Southern China.

**Methods:**

A retrospective cross-sectional study (*n* = 361) was conducted using a random sample of cases from a primary care database registry of two CHSC in Guangzhou city, Guangdong province, China. The sample consisted of community-dwelling older adults aged 60 years and over who receive free annual medical examinations provided by the Chinese government. Data collected included biomarkers for chronic diseases, medical history, levels of physical activity, and lifestyle risk factors.

**Results:**

There were 361 cases [mean age 74.65 (SD = 5.61) years] included in the sample (from total registry records *n* = 6,351). The top five chronic diseases were hypertension (55.68%), being overweight or obese (43.77%), hyperuricemia (40.60%), fatty liver disease (34.35%) and hypercholesterolemia (17.17%). Being overweight or obese was significantly associated with having fatty liver disease (OR = 1.22, 95% CI 1.05–1.41), higher WC was significantly associated with having hypertension (OR = 1.05, 95% CI 1.02–1.07), hyperuricemia (OR = 1.04, 95% CI 1.01–1.07), dyslipidemia (OR = 1.09, 95% CI 1.03–1.16), and fatty liver disease (OR = 1.22, 95% CI 1.05–1.41). Smoking was significantly associated with having hyperuricemia (OR = 2.40, 95% CI 1.13–5.07).

**Conclusion:**

Data collected via medical examination identified the top five chronic diseases among older adults of Guangzhou city, China. Lifestyle risk factors are significantly associated with chronic diseases. Findings from the study will inform future design and evaluation of targeted, new services for these older adults. Further research to evaluate lifestyle interventions that can improve the health of older adults living with chronic disease is required.

**Trial registration:**

Ethical approval ([2022]02-014-01) was obtained from the Health Research Ethics Committee of the Third Affiliated Hospital of Sun Yat-Sen University, Guangzhou, China. The study was registered on the Chinese Clinical Trial Registry Centre (registry number: ChiCTR2200066750).

## Introduction

Chronic diseases (defined by the National centre for chronic disease prevention and health promotion: as “conditions that last 1 year or more and require ongoing medical attention or limit activities of daily living or both”) are recognised as a worldwide public health problem (1, 2). Annually, an estimated 41 million people die from major chronic diseases, accounting for 74% of all deaths worldwide, with 77% of these deaths occurring in low- and middle-income countries (1). In China, 80% of deaths and 70% of disability-adjusted life-years lost are attributable to chronic diseases (2). The prevalence of chronic diseases increases with ageing due to ageing changes in body systems, genetics (non-modifiable risk factors), and lifestyle factors, including smoking, diet, and exercise patterns (modifiable risk factors) (3, 4).

Approximately 50% of older adults in China have at least one chronic disease, and 15% have at least two (5). Older adults (60 years and over) living with chronic diseases have significantly more outpatient visits, longer hospital stays, and extra health care costs, and greater levels of functional disability and decreased quality of life compared to those without chronic disease (6). This has led to an escalating social and economic burden of chronic diseases (7). Therefore, managing chronic diseases is a high priority in China as it has been estimated that the older population (60 years and over) in China will reach 200 million (30% of the total population). The world’s older population will reach 2.1 billion by 2050 (8, 9).

According to the World Health Organisation (WHO), the four most common chronic diseases are cardiovascular diseases (heart attacks and strokes), cancers, chronic respiratory diseases (asthma and chronic obstructive pulmonary disease) and diabetes (1). However, studies in China reported some variation in this prevalence with hypertension and high cholesterol being the most prevalent diseases in older adults (2, 10). Recent research in China reported that the three most prevalent chronic diseases among older community-dwelling adults were hypertension, diabetes, and hypercholesterolemia, with cancers only accounting for 2.5% of chronic diseases. This large multi-centre study included data from 31 Chinese provinces, but data were collected using self-reported questionnaires rather than bio-markers, which may have reduced the reliability and validity of these results (11). Other studies have also found variation within different provinces in China compared to the WHO prevalence (10). However, evidence for prevalence of chronic diseases using valid and reliable diagnosis is still a knowledge gap in southern China.

Addressing modifiable lifestyle risk factors is a key strategy for reducing the incidence of chronic diseases (12). Globally, tobacco causes over eight million deaths yearly; excess salt/sodium intake leads to 1.8 million deaths; insufficient physical activity causes 830,000 deaths; and these behavioural risk factors are responsible for to 19% of all causes of mortality worldwide (1). An international study conducted in six middle and low-income countries, including China and India, found that behavioural risk factors, including moderate and high-fat diets, tobacco use, insufficient physical activity, and the harmful use of alcohol, were independent risk factors for chronic diseases in older adults (13, 14). This study reported that the prevalence of current daily smokers in China among adults aged 60–69 years was only lower than the prevalence in India, and heavy alcohol consumption was highest in China compared with other countries. As a result, hypertension (64%) among adults aged 60–69 years was the most prevalent chronic disease in China (14). The impact of lifestyle on the prevalence of chronic diseases is also an increasing problem in high-income countries, such as the. An UK cross-sectional survey reported that older females (65 years and over) were more likely to report insufficient levels of physical activity, and older males were more likely to consume harmful levels of alcohol (15).

The WHO highlights that a healthy lifestyle is a way of living that lowers the risk of becoming seriously ill or dying early (16). Adults with multiple lifestyle risk factors tend to have more chronic diseases than those with one lifestyle risk factor (15). Therefore, the development of a risk factor profile for chronic diseases can provide essential information for government to identify the future burden of chronic diseases and develop policies that aim to improve communities’ lifestyle choices (14). For instance, eating foods high in fat, sugar, and salt, such as fast food, is one of the main reasons for a rise in the prevalence of obesity. In response, the United Kingdom government developed a sugar tax in 2018 to reduce the household purchasing of high-sugar content drinks and introduced policies that restrict the proportion and density of fast-food outlets in local communities (17–19). However, in China, there is a lack of high-quality evidence regarding the prevalence of chronic diseases and associated lifestyle risk factors. Epidemiological studies in China to date have used self-report surveys rather than biomarkers, hence there is limited robust evidence identifying the association between chronic diseases and lifestyle risk factors in Chinese older adults. Additionally, studies that have evaluated chronic disease patterns among older adults in the southern cities of China are sparse.

Community health services centers (CHSCs) in China are the key primary health institutions for providing medical and public health services, including daily living assistance, rehabilitation, medical care, psychological services, and free health care examinations for older adults aged 60 year and over (20). There are over 34,000 CHSC in China. These CHSC provide services according to standard Chinese government regulations. The centres also focus on chronic diseases prevention and control in their communities, as an essential strategy of Chinese national chronic diseases management (18). Lifestyle risk factors are a potential area of intervention for risk reduction that can be addressed by CHSC programs. There is an increasing social, health, and economic burden caused by chronic diseases in China. However, limited studies have evaluated the prevalence of chronic diseases based on individual clinical examination results with biomarkers that accurately confirm diagnosis, such as fasting blood glucose measurements (21). To obtain accurate data on this problem, it is necessary to conduct individual health examinations identify patterns of chronic diseases and lifestyle risk factors among older Chinese adults. These data are collected from older adults who attend CBC for an annual health examination. The aim of the study was to evaluate the association between chronic diseases and lifestyle risk factors among community-dwelling older adults living in Guangzhou, China. Findings from the study were expected to inform future design and evaluation of new CHSC services for these older adults.

## Methods

### Ethics approvals and trial registration

Ethical approval ([2022]02-014-01) was obtained from the Health Research Ethics Committee of the Third Affiliated Hospital of Sun Yat-Sen University, Guangzhou, China for a de-identified database to be provided to the researchers through a secure link. All personal information (such as name, address, and national identification number) of subjects was removed prior to the dataset being delivered to the research team. The study was registered on the Chinese Clinical Trial Registry Centre (registry number: ChiCTR2200066750). The study was reported according to the Reporting of studies Conducted using Observational Routinely collected health Data (RECORD) Statement. This statement is an extension to the Strengthening the Reporting of Observational Studies in Epidemiology (STROBE) Statement: guidelines for reporting observational studies (see Supplementary Table S10) (22). To protect participants’ privacy, all identifiable information was removed before the database was provided to the research team. This process ensured that individual participants could not be identified from the dataset. A waiver for obtaining individual consent was thus provided.

### Study setting

Data were collected from two communities in Guangzhou, Guangdong province, China. These two communities are in one district in Guangzhou City (total population 2,238 million) which has an advanced economy and over 174 thousand (8.6%) of the population is aged 60 years and over (23). Adults 60 years and older in Guangzhou (female 52.9%, male 47.0%) are mostly retired office workers (42.9%) or civil servants (30.7%) with a few traders and householders (23).

### Study design and population

The study used data from a primary care database in the Guangzhou area. Data were collected from *n* = 361 (162 [44.9%] males) adults aged 60 years and over in Guangzhou communities, who receive free annual medical examinations provided by the Chinese government at CHSC. A retrospective cross-sectional study design was used with a random sample of cases from the database registry.

#### Sample size

The sample size for the present study (*n* = 361) was calculated based on the number of cases required to obtain a representative sample of a population, using sample size calculations provided by Krejcie and Morgan (24). A sample of *n* = 361 cases met the requirements of being sufficiently representative of the total registry of 6,351 records.

A three-stage random sampling method was used to select cases for analysis (25). In the first stage of sampling, two Chinese Community Health Service Centres (CHSC) (both in Tianhe district) provided the number of all medical examinations for adults aged 60 years and over that were completed between the 1^st^ January 2020 and 31^st^ December 2021. All cases were reviewed by the CHSC, and if they contained missing data, the case was excluded, (leaving 6,351 cases in total). Secondly, a sample of the complete cases was selected using a sequence created with a random number generator in Excel to identify 361 random numbers between 1 and 6,351. The sequence was generated by a researcher in Australia who was not involved in data collection or management of the health database in Guangzhou. Thirdly, the sequence was then sent to the researcher in China who provided the numbers to the database registry. The registry then provided the data for the allocated numbers which matched the relevant case record. The registry followed the number sequence provided and had no involvement in selection of case records. The data manager then transferred the de-identified data through secure online transfer to the University. The researchers in China and Australia were blind to any data or information cases in the database until after the allocation of the cases, when they were provided with the data for each included case record.

### Variables: Assessment of chronic diseases

Eight chronic diseases and health conditions were measured through the medical examinations which were performed by nurses working in the CHSC with diagnoses confirmed by the doctor after blood tests, including hypertension, hyperglycemia, dyslipidemia, hyperuricemia, fatty liver disease, obesity, and left ventricular hypertrophy. The following diagnostic criteria were used:

1. Hypertension: systolic blood pressure (SBP) ≥ 130 mm Hg (26).
2. Hyperuricemia: uricemia (UA), indicating impaired kidney function: male >420 μmol/L, female >350 μmol/L. (27)
3. Hyperglycemia (diabetes): fasting blood glucose (FBG) ≥ 7.0 mmol/L; based on the WHO diagnostic criteria for diabetes (28).
4. Hypercholesterolemia: triglyceride (TG) ≥2.3 mmol/L or total cholesterol ≥6.2 mmol/L (TC); based on the guidelines for the prevention and treatment of dyslipidemia or adults in China (28).
5. Dyslipidemia: low density lipoprotein (LDL) ≥4.1 mmol/L or high-density lipoprotein (HDL) <1.0 mmol/L. (28)
6. Overweight: 28 kg/m^2^ > body mass index (BMI) ≥ 24 kg/m^2^, Obesity: ≥28 kg/m^2^ (27).
7. Fatty liver disease: diagnosis (yes/ no) according to the ultrasound, results provided by doctors who conducted the test.
8. Heart disease (left ventricular hypertrophy): diagnosis (yes / no) according to ECG, results provided by a cardiologist who reviewed the ECG results.

Other data provided for cases were gender, age, waist circumference (WC), lifestyle risk factors (smoking and alcohol status) and levels of physical activity.

### Assessment of lifestyle risk factors and physical activity

Data related to lifestyle habits were collected during the older adult’s annual medical examinations by trained nurses interviewing the older adult. Questions enquired about smoking status (yes or no) and amount and type of physical activity. Both alcohol consumption and physical activity levels included four levels and, excessive consumption (more than 14 standard drinks per week for men and more than 7 standard drinks per week for women), moderate consumption (up to 14 drinks 7 standard per week for men and up to 7 standard drinks per week for women), occasional consumption (less than 4 standard drinks per week), non-drinkers (no alcohol consumption) (29). Physical activity was recorded by asking open-ended questions about the type, frequency and amount of activity, e.g., how many times per week, how long each session. Physical activity was subsequently classified using four levels, 0 = no activity, 1 = low intensity activity, 2 = moderate intensity activity, and 3 = vigorous intensity activity. This classification was undertaken based on the exercise mode and time undertaken weekly as framed by the WHO guidelines for physical activity for older adults (30). Physical activities undertaken for less than 150 min weekly and of low intensity (such as Tai chi or walking) were classified as low intensity activity. Physical activities undertaken for at least 150 min a week (for example, 30 min a day, 5 days a week) of low intensity exercises were classified as moderate intensity activity. If the activities were 75 min a week of high intensity exercises (such as bicycling, hiking, jogging, square dancing, strength training or running), the physical activities were classified as vigorous activity (31). Dietary intake was not recorded however measurements of waist circumference and BMI were treated as surrogate indicators of diet as a lifestyle risk factor, since diet is known to significantly impact weight management (32, 33).

### Assessment for cardiovascular diseases risk

The risk of cardiovascular diseases (CVD), including all heart, stroke, and blood vessel diseases, was measured by using the CVD risk calculator suggested by the Australian Chronic Disease Prevention Alliance (ACDPA) guidelines (34). Absolute risk is categorised according to the influence of risk factors working together, including, gender, age, systolic blood pressure, smoking status, total cholesterol, HDL cholesterol, diabetes history and left ventricular hypertrophy history, and results can be communicated to patients, as the risk of developing cardiovascular heart disease in the next 5 years being low (< 10%), moderate (10–15%) or high (> 15%). Since this assessment is only suitable for people aged 35–74 years, only cases with ages from 60 to 74 years in this study underwent a risk score calculation. The calculation of CVD risk was conducted through entering data for each case into the CVD check official website.^1^

### Statistical analysis

Lifestyle risk factors (smoking and alcohol) were coded as indicator variables (absence/presence of related condition). Hypertension, hyperuricemia, hyperglycemia, hypercholesterolemia (TC), hypercholesterolemia (TG), dyslipidemia (LDL), dyslipidemia (HDL), fatty liver disease, and left ventricular hypertrophy, were coded as indicator variables (absence/presence of disease) based on the diagnostic criteria described above; BMI was categorised into underweight, normal, overweight, and obesity groups.

Data were summarised using descriptive statistics, with frequency distributions for categorical data and means and standard deviations for normally distributed continuous data (normality tested using Shapiro Wilk tests). Group comparisons of continuous variables were made using independent t-tests and one-way ANOVA. Categorical comparisons were made using Pearson’s Chi-squared tests.

Dependent variables, including indicators of disease hypertension, hyperuricemia, hyperglycemia, hypercholesterolemia (TC), hypercholesterolemia (TG), dyslipidemia (HDL), dyslipidemia (LDL), fatty liver diseases and Left ventricular hypertrophy, were evaluated using logistic regression models. Independent variables included BMI, WC, smoking, alcohol, physical activity, and blood indicators that are known to be clinically associated with the dependent variables. All the models were adjusted for age and gender. Candidate variables from univariable logistic regression models were entered into multivariable logistic regression models. Covariate effects from logistic regression analyses were summarised using odds ratios (OR) and 95% CIs were used to summaries logistic regression results. Covariate effects from logistic regression analyses were summarised using odds ratios (OR) and 95% CIs were used to summarise logistic regression results. Stata version 16 software (StataCorp, College Station, Texas) was used to complete all analyses. A *p* value <0.05 (two-tailed) indicated statistical significance.

## Results

### General characteristics of subjects

Characteristics of the sample are presented in Table 1. The database (*n* = 361 cases) consisted of 162 (44.88%) males and 199 (55.12%) females. The mean age of the sample was 74.65 (SD = 5.61) years and differed significantly between males and females (*p* = 0.006). The total mean BMI was 23.67 (SD = 3.16) (kg/m^2^). The mean WC was 83.59 (SD = 8.84) cm and differed significantly between males and females (*p* < 0.001). There were no significant differences in medical history [diabetes (*p* = 0.660), HBP (*p* = 0.355), or physical activity behaviours (*p* = 0.087)] between genders.

**Table 1:** Characteristics of the sample.

Variables	Category	Whole Cohort	Male	Female	*p*-value
		*n* = 361/Mean ± SD ^a^	*n* = 162 (44.88%)/Mean ± SD ^a^	*n* = 199 (55.12%) Mean ± SD ^a^	
Age (year)^a^		74.65 ± 5.61	75.49 ± 5.78	73.97 ± 5.39	0.006^#^
BMI (kg/m^2^)^a^		23.67 ± 3.16	23.76 ± 3.25	23.60 ± 3.10	0.535^#^
WC (cm)^a^		85.61 ± 8.84	88.09 ± 9.28	83.59 ± 7.95	<0.001^#^
Smoking (yes)^b^		51 (14.13)	51 (31.48)	0	<0.001^&^
Alcohol consumption^b^	None	305 (84.39)	142 (87.65)	163 (95.88)	0.294
	Occasional	37 (10.25)	14 (8.64)	23 (11.56)	
	Excessive	19 (5.26)	6 (3.70)	13 (6.53)	
History of diabetes (yes)^b^		83 (22.99)	39 (24.07)	44 (22.11)	0.660^&^
History of HBP (yes)^b^		173 (47.92)	82 (50.62)	91 (45.73)	0.355^&^
Physical activity levels ^b^	None	230 (63.71)	28 (17.28)	43 (21.61)	0.324^&^
	Low	60 (16.62)	23 (14.19)	37 (18.59)	
	Moderate	218 (60.39)	104 (64.19)	114 (72.36)	
	High	12 (3.32)	7 (4.32)	5 (2.51)	

a = mean (SD for continuous variables only); b = n (%); Physical activity behaviors = (Yes = high intensity physical activity and median intensity physical activity, No = low intensity physical activity and no physical activity); ^#^Conducted using T test; ^&^Conducted using Chi^2^ test.

A CVD risk score was calculated for the 202 cases in the database (53.96%) who were less than 74 years of age. Among these cases, 79 older adults (39.11%) had a low CVD risk score (< 10%), 24 older adults (11.88%) had a moderate CVD risk score (10–15%), and 99 older adults (49.00%) had a high CVD risk score (> 15%).

### Prevalence of chronic diseases

**Figure 1:**
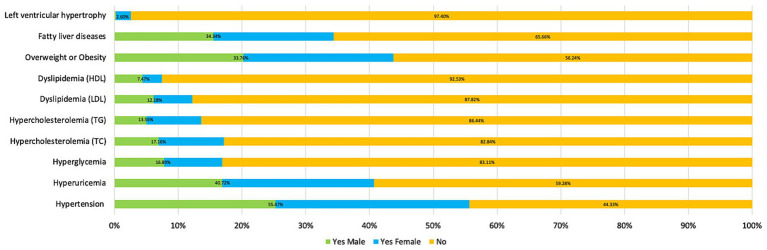
The prevalence of chronic diseases and health conditions by gender.

More than a half the sample (205/361, 56.79%) were classified as having chronic disease multimorbidity (presence of two or more chronic diseases), 27.42% (99/361) of the sample had one chronic disease and 15.78% (57/361) had no chronic disease. Table 2 presents the prevalence of chronic diseases and health conditions of the sample, as indicated by medical test results. Figure 1 compares total prevalence of chronic diseases and health conditions and the prevalence for males and females. The top five chronic diseases were hypertension (SBP) (55.68%), being overweight or obese (BMI) (43.77%), hyperuricemia (UA) (40.60%), fatty liver disease (34.35%) and hypercholesterolemia (TC) (17.17%). Prevalence of comorbidities, including hypertension and overweight or obesity, hypertension and hyperuricemia, and overweight or obesity (n/%) and hyperuricemia compared between genders (see Supplementary Table S1).

**Table 2:** Medical test results compared between genders.

Variables	Whole Cohort		Male		Female		*p*-value
	*n* = 361 (100%)	Mean ± SD^k^	*n* = 162 (44.88%)	Mean ± SD^k^	*n* = 199 (55.12%)	Mean ± SD^k^	
Hypertension (SBP^a^) (yes)	201 (55.68)	132.06 ± 18.34	91 (56.17)	133.68 ± 18.53	110 (55.28)	131.06 ± 18.15	0.174^#^
Hyperuricemia (UA^b^) (yes)	147 (40.60)	368.01 ± 89.22	61 (37.00)	397.50 ± 87.61	86 (43.21)	344.01 ± 83.32	0.285^#^
Hyperglycemia (FBG^c^) (yes)	61 (16.90)	6.30 ± 1.67	28 (17.28)	6.35 ± 1.60	33 (16.58)	6.26 ± 1.74	0.162^#^
Hypercholesterolemia (TC^d^) (yes)	62 (17.17)	5.23 ± 1.09	25 (15.43)	5.08 ± 1.09	37 (18.59)	5.36 ± 1.09	0.011^#^
Hypercholesterolemia (TG^e^) (yes)	49 (13.57)	1.53 ± 0.89	18 (11.11)	1.46 ± 0.89	31 (15.58)	1.58 ± 0.89	0.029^#^
Dyslipidemia (LDL^f^) (yes)	44 (12.19)	3.13 ± 0.90	22 (13.58)	3.11 ± 0.92	22 (11.06)	3.16 ± 0.89	0.587^#^
Dyslipidemia (HDL^g^) (yes)	27 (7.48)	1.47 ± 0.37	16 (9.88)	1.36 ± 0.33	11 (5.53%)	1.56 ± 0.38	<0.001^#^
Overweight or obesity^h^ (yes)	158 (43.77)	23.67 ± 3.16	73 (45.06)	23.76 ± 3.25	85 (42.71%)	23.59 ± 3.10	0.655^#^
Fatty liver disease^I^ (yes)	124 (34.35)	n/a	56 (34.57)	n/a	68 (34.17)	n/a	0.937^&^
Left ventricular hypertrophy^j^ (yes)	10 (2.77)	n/a	1 (0.62)	n/a	9 (4.52)	n/a	0.025^&^
Multi-comorbidities (*n*/%)^k^ (yes)	204 (56.51)	n/a	92 (56.79)	n/a	112 (56.28)	n/a	0.923^&^

a = (SBP ≥ 130); b = (UA: male > 420 μmol/L, female > 350 μmol/L); c = (FBG ≥ 7 mmol/L); d = (TC ≥ 6.2 mmol/L); e = (TG ≥ 2.3 mmol/L); f = (LDL ≥ 4.1 mmol/L); g = (HDL<1.0 mmol/L); h = (overweight: 28 kg/m^2^ ≥ BMI > 24 kg/m^2^, obesity: BMI > 28 kg/m^2^); i = (light fatty liver, mild fatty liver and severe fatty liver disease); j = (Left ventricular hypertrophy diagnosed by ECG) k = mean (SD for continuous variables only); k = (have two or more than two chronic diseases, including hypertension; hyperuricemia; hyperglycemia; hypercholesterolemia; dyslipidemia; and fatty livers). ^#^Conducted using T test; ^&^Conducted using Chi^2^ test.

### Logistic regression analysis for outcomes of chronic diseases

The final multivariable models for outcomes of chronic diseases are presented in Table 3. Full univariable and multivariable models for all outcomes are presented in the supplementary file (Supplementary Tables S2–S9).

**Table 3:** Logistic regression analysis of influencing factors for chronic diseases and health conditions.

Logistic regression	Outcome	Predictor	Multivariable*		
			OR	95% CI	*p*
Model 1	Hypertension	WC (cm)	1.05	1.02, 1.07	0.001
		Hyperuricemia	1.62	1.03, 2.53	0.036
Model 2	Hyperuricemia	WC (cm)	1.04	1.01, 1.07	0.006
		Smoking	2.40	1.13, 5.07	0.022
		Hypertension	1.66	1.03, 2.68	0.039
		Hypercholesterolemia (TC)	2.21	1.24, 3.94	0.007
		Hypercholesterolemia (TG)	2.79	1.29, 6.01	0.009
		Dyslipidemia (HDL)	4.12	1.29, 13.15	0.017
Model 3	Hyperglycemia	Hyperuricemia (UA)	0.47	0.25, 0.88	0.019
		Dyslipidemia (TG)	3.37	1.62, 6.98	0.001
Model 4	Hypercholesterolemia (TC)	Dyslipidemia (LDL)	2.78	7.02, 11.02	<0.001
Model 5	Hypercholesterolemia (TG)	Dyslipidemia (LDL)	3.91	1.74, 8.76	0.001
		Dyslipidemia (HDL)	6.19	2.29, 16.67	<0.001
		Fatty liver diseases	6.83	3.30, 14.14	<0.001
Model 6	Dyslipidemia (LDL)	WC (cm)	1.09	1.03, 1.16	0.005
		Hypercholesterolemia (TC)	3.89	8.64, 17.51	<0.001
		Hyperuricemia (UA)	1.99	1.04, 3.83	0.038
Model 7	Dyslipidemia (HDL)	Hyperuricemia (UA)	4.44	1.64, 12.05	0.003
		Hypercholesterolemia (TG)	5.32	1.76, 16.10	0.003
		Fatty liver disease	2.86	1.01, 8.10	0.047
Model 8	Fatty liver disease	Overweight or obesity	1.22	1.05, 1.41	0.009
		WC (cm)	1.06	1.01, 1.11	0.020
		Hypercholesterolemia (TG)	7.33	3.49, 15.38	<0.001

* = all analyses adjusted by age and gender. UA = uric acid; FBG = fasting blood suger; TC = total cholesterol; TG = triglycerides; LDL = low-density lipoprotein; HDL = high-density lipoprotein.

*Model 1* (hypertension) demonstrated that higher WC and having hyperuricemia were significantly associated with having hypertension. *Model 2* (hyperuricemia) showed that higher WC, smoking, having hypertension, hypercholesterolemia (TC and TG), and dyslipidemia (HDL) were significantly associated with having hyperuricemia. *Model 3* (hyperglycemia) demonstrated that having dyslipidemia (TG) was positively significantly associated with having hyperglycemia, with older adults who had dyslipidemia (TG) being over three times more likely to have hyperglycemia. *Model 4* (hypercholesterolemia) demonstrated that having dyslipidemia (HDL) was positively significantly associated with having hypercholesterolemia (TC). *Model 5* (hypercholesterolemia) caused by high TG: showed that higher levels of HDL and LDL, were significantly associated with having hypercholesterolemia (TG). *Model 6* (dyslipidemia) caused by high LDL showed that higher TC and higher WC and having hyperuricemia was significantly associated with dyslipidemia (LDL). *Model 7* (dyslipidemia) caused by lower HDL demonstrated that having higher UA, TG and being diagnosed by fatty liver diseases were significantly associated with having dyslipidemia (HDL). *Model 8* (fatty liver diseases) showed that having obesity or overweight and higher levels of WC and having hypercholesterolemia (TG) were significantly associated with having fatty liver disease.

## Discussion

Guangzhou city is the capital city of Guangdong province, in the southern part of China. The proportion of the population that is 60 years and over is 11.41% (2.1 million), which is below the Chinese national average of 18.7% (264.0 million). However, Guangzhou city still meets the United Nations criteria for an ageing city (23). This current study evaluated the association between lifestyle risk factors and prevalence of chronic diseases among older adults in Guangzhou by using diagnostic chronic disease biomarkers collected during older adults’ annual free medical examination (35). Therefore, results of this study have better validity and reliability when compared with previous studies, which reported the association between lifestyle risk factors and prevalence of chronic disease using self- reported data collected by questionnaires.

Findings demonstrated that the top five chronic diseases in Guangzhou city were hypertension, being overweight or obese, hyperuricemia, fatty liver disease and hypercholesterolemia (TC). Over half the sample had hypertension and over 40% were overweight or obese. These results contrasted with a previous study reported by Pan et al. (36) conducted in 2009 that collected data using self-reported surveys. Pan et al. found that the top five chronic diseases in Guangzhou city were hypertension, type 2 diabetes, chronic rhinitis, lipoprotein metabolism disorders, and coronary heart disease (36). These differences between the previous study in Guangzhou and our study may be due to our methods of collecting these data, but also could reflect the rapid development of the economy and associated changes in older adults’ lifestyles over the past 15 years (36). The previous study noted that while the prevalence of smoking had decreased the prevalence of overweight and obesity increased in Guangzhou (36). A recent national Chinese study also reported that between 1993 and 2018 two lifestyle risk factors (drinking and obesity) increased in prevalence, even though physical inactivity and smoking decreased. Therefore, lifestyle risk factors are increasingly affecting the increase in chronic diseases in China (37). Findings compare with Shanghai, another well-developed city in China, where the top three chronic diseases among older adults have been reported in 2022 as being hypertension (65.4%), being overweight or obese (53.8%), and hypercholesterolemia (TC) (29.6%) (24). However, by contrast, in the UK, arthritis (62.6%), hypertension (55.9%), respiratory diseases (24.4%), cancer (23.7%), and diabetes (21.6%) are the five most prevalent chronic diseases in older adult populations (18).

Over half of our population had hypertension based on their medical examination, and this increased with age. These results compare with a large Chinese national study of over 175,000 cases in 31 provinces, which reported the prevalence of hypertension was 58.3% (11). However, these data were collected 10 years ago using a self-reported questionnaire, which could be less reliable than using medical examination results. The prevalence of hypertension [the diagnostic criteria of hypertension (is SBP ≥ 140)] for the population aged 60 years and over is over 60% in Australia and 77.9% in South Africa, which is higher than the prevalence found in our study (38, 39). However recently published clinical practice guidelines for the management of hypertension in China (2022), re-classified the diagnostic criteria for hypertension from SBP ≥ 140 mmHg to SBP ≥ 130 mmHg (26), which may impact these comparisons.

Hyperuricemia prevalence in our population was 40.6%. Hyperuricemia is associated with the occurrence and development of chronic kidney disease (CKD), hypertension, diabetes, metabolic syndrome, and obesity (40). The prevalence of hyperuricemia in Shanghai in the eastern part of China, where researchers also diagnosed chronic diseases in older adults by using blood tests, ranged from 22 to 27% which contrasts with our findings (41). Findings also contrast with the prevalence of hyperuricemia among older adults in Poland (28.2% of women and 24.7% of men) and Australia (18.4%) (38, 40). However, our findings are similar to a recent study also conducted in Guangdong province, that found a hyperuricemia prevalence of between 31.5 and 41.7% for older community-dwellings adults (42).

Our results showed that the prevalence of hyperglycemia was 16.9% among older adults in Guangzhou. These results are a lower than the average worldwide prevalence of older adults with hyperglycemia, which is reported as 19.3% (43). A very large Chinese multi-centre epidemiological study similarly reported that the prevalence of hyperglycemia among older community-dwelling adults in China was 19.4%. The prevalence of hypercholesterolemia (TC 17.1%; TG 13.5%) was much lower than reported in previous studies conducted in other large Chinese cities [(Shanghai: TC 35.1%; TG 29.6%) (Tianjin: TC 60.5%; TG 38.6%)] (27, 44). The prevalence of fatty liver disease (34.3%) was also lower than that reported by previous studies in other cities (47.9% in Shanghai, 44.4% in Wuhan) (45, 46).

Lifestyle changes are an important focus of preventive health efforts worldwide to reduce this burden of chronic disease among older adults (1). BMI and WC are a known surrogate measure of dietary lifestyle and significant risk factors for increased morbidity and mortality (32, 33). These risk factors also have a negative impact on quality-adjusted life years in older adults (32, 33). Our findings showed that being overweight and obese, as categorized by BMI, was significantly associated with fatty liver disease, which is similar to findings from a Chinese study with a larger sample size that enrolled a cohort aged 45 years and older living in the community (47). Having a larger waist circumference was associated with having hypertension, hyperuricemia, dislipidemia, and fatty liver disease. Previous research has shown that each standard deviation higher of waist circumference is associated with 1.3 times higher prevalence of hypertension (48). We found that higher, WC and TG were significantly associated with having fatty liver disease. Liver disease is still one of the top five causes of death in the world, and fatty liver disease, has gradually replaced viral hepatitis as the world’s most prevalent liver disease (49). In addition to affecting liver tissue, many studies have confirmed that fatty liver is often combined with hyperlipidemia, diabetes, hyperuricemia, hypertension, and other chronic diseases, suggesting that these diseases may be an important cause of fatty liver (46).

Tobacco use is the leading preventable cause of chronic diseases due to the harmful chemicals it contains, which induces inflammation and oxidative stress, contributing to the development of cardiovascular diseases (50). Our findings demonstrated that smoking was a lifestyle risk factor that was significantly associated with having hypertension and hyperuricemia. These results were consistent with a longitudinal study conducted in Finland that found 43% of middle-aged normotensive men who smoked developed hypertension (51).

The low prevalence of chronic diseases in Guangzhou compared with some other cities in China and counties could be partially ascribed to better lifestyle behaviours. There were fewer smokers (14.13%) and lower alcohol consumption (15.51%). The prevalence of older adults engaged in physical activities (80.33%) was higher compared to other cities in China (5), however physical activity was only self-reported. Our cohort consisted of mainly retired older adults and retirement in China is known to be associated with significant decline in physical activity, indicating that more public health efforts to promote healthy ageing for this cohort is required (52). The prevalence of being overweight and obese in our study was much lower than other cities in China (49.7% in Shanghai, 64.8% in Tianjin, 51.67% in Wuhan) (27, 38, 44). The incidence of chronic diseases, including hypertension, in southern China has been reported to be lower than in northern China, due to lower dietary salt intake (36). Moreover, differences in the prevalence of chronic diseases between provinces in China may be due to differences in alcohol intake. A Chinese national study reported that the prevalence of alcohol intake is highest in Shandong in middle China and Heilongjiang in northeast China, which may be related to levels of education and family income (53).

### Strengths and limitations

A major strength of this study was that we evaluated the prevalence of chronic diseases using biomarkers obtained by medical and laboratory examinations, which improves the reliability of the results. This study also used a multi-centre design by collecting data from two CHSCs in one district, which aimed to improve the representativeness of the results for other CHSCs in Southern China. Moreover, our dataset contained minimal missing data with evidence-based sampling strategy. Findings of this study can be used to inform further research and evaluation of new community-based services for older adults living in China.

Limitations of the study included that the recorded medical history for each case was limited. Therefore, the presence of diseases such as dementia, stroke, and musculoskeletal diseases were not able to be included in the data set and used in the analyses, for example to identify the influence of multimorbidity. Secondly, lifestyle-risk factors data including alcohol consumption, smoking history, and physical activity levels were collected by a trained nurse, but using a non-validated questionnaire, which limited the information provided and therefore reduces the reliability of the results. Physical activity was self-reported, and it was not possible to categorise cases rigorously, other than using a broad scale. Analyses may not have been sensitive to this variable, as physical activity is known to be associated with chronic diseases such as hypertension and hyperglycemia, as demonstrated in other large studies in China (54). Other risk factors not measured included dietary intake, socioeconomic status and education which are known to be associated with chronic disease (55). We did measure WC and BMI and used this as an indicator of diet. A key limitation was that this study used an observational and cross-sectional design so associations identified might be difficult to interpret (56). These findings are not casual, but can be used to support further research, including designing and evaluating new services Southern China’s CHSC.

## Conclusion

Data collected via annual medical examinations identified the top five chronic diseases in a large city in Southern China and found that lifestyle risk factors are significantly associated with hypertension, hyperuricemia, dyslipidemia, and fatty liver disease. It is anticipated that our findings will inform community health services in Southern China to design tailored community-based exercise programs and other lifestyle programs for older adults, as the cohort represents those population who attend CHSCs. These CHSCs have been established by the Chinese government as the key primary health institutions for promoting health and wellbeing. This role is undertaken through providing medical and public health services, including for older adults. Therefore, providing accurate and reliable data about the older population who attend CHSCs is essential to guide further government policy and practice in these centres. Further research that investigates the association between chronic diseases and lifestyle risk factors in China should include valid and reliable measurements of lifestyle risk factors. Further research should evaluate the effectiveness of interventions that improve older adults’ health in China by addressing lifestyle factors, to reduce the prevalence of chronic diseases.

## Data Availability

The original contributions presented in the study are included in the article/Supplementary material, further inquiries can be directed to the corresponding authors.

## Footnotes

^1^ https://www.cvdcheck.org.au/
