# Supplementary material for: Association between chronic diseases and lifestyle risk factors among community-dwelling older adults: a retrospective cross-sectional Chinese population-based study
Source: Front Public Health. 2025 Mar 5;13:1435385. doi: 10.3389/fpubh.2025.1435385 (PMC11919672; doi:10.3389/fpubh.2025.1435385)
Supplement: Supplementary file 1 [file Supplementary_file_1.docx]

**SUPPLEMENTARY FILE**

**Title** Association between chronic diseases and lifestyle risk factors among community-dwelling older adults: a retrospective cross-sectional Chinese population-based study

**Authors**

XIN Wei^1,2,6^ PhD candidate, XU Dan^1,3,4^ PhD, DOU Zulin^2^ PhD, JACQUES Angela^5^ MBiostat, UMBELLA Josephine^6^ BSc, FAN Yuling^7^ MBBS, ZHANG Longsheng^8^ MBBS, YANG Haiwen^7^ MBBS, CAI Hong^8^ MBBS, HILL Anne-Marie^6^ PhD

^1^ Curtin University, Curtin Medical School, Perth, Western Australia, Australia

^2^ The Third Affiliated Hospital of Sun Yat-Sen University, Rehabilitation Medicine department, Guangzhou, Guangdong, China

^3^ Curtin University, Curtin School of Population Health, Faculty of Health Sciences, Perth, Western Australia, Australia

^4^ The First Affiliated Hospital of Sun Yat-Sen University, General Practice department, Guangzhou, Guangdong, China

^5^ The University of Notre Dame Australia, Institute for Health Research, Fremantle, Western Australia, Australia

^6^ The University of Western Australia, School of Allied Health, Perth, Western Australia, Australia

^7^ Guangzhou Tianhe District Shipai street community health service center, Guangzhou, Guangdong, China

^8^ Guangzhou Tianhe District Linhe street community health service center, Guangzhou, Guangdong, China

**Corresponding author 1:** Wei Xin

**Corresponding author Email:** wei.xin1@postgrad.curtin.edu.au

**Telephone number**: +86 18819815060

**Corresponding author 2:** Zulin Dou

**Corresponding author Email:** douzul@163.com

**Supplementary file**

| **Table** | **Item** |
| --- | --- |
| Table S1 | Prevalence of comorbidities compared between genders |
| Table S2 | Logistic regression analysis of influencing factors of Hypertension |
| Table S3 | Logistic regression analysis of influencing factors of Hyperuricemia |
| Table S4 | Logistic regression analysis of influencing factors of Hyperglycemia |
| Table S5 | Logistic regression analysis of influencing factors of Hypercholesterolemia (TC) |
| Table S6 | Logistic regression analysis of influencing factors of Hypercholesterolemia (TG) |
| Table S7 | Logistic regression analysis of influencing factors of Dyslipidemia (LDL) |
| Table S8 | Logistic regression analysis of influencing factors of Dyslipidemia (HDL) |
| Table S9 | Logistic regression analysis of influencing factors of Fatty liver disease |
| Table S10 | STROBE Statement—Checklist of items that should be included in reports of cross-sectional studies |

Table S1 Prevalence of comorbidities compared between genders

| Variables | Whole Cohort | Male | Female | p-value |
| --- | --- | --- | --- | --- |
|  | n=361(100%) | n =162 (44.88%) | n =199 (55.12%) |  |
| Hypertension and Overweight or obesity (n/%) | 96 (26.59) | 47 (29.01) | 49 (24.62) | 0.348 |
| Hypertension and Hyperuricemia (n/%) | 94 (26.04) | 37 (22.84) | 57 (28.64) | 0.211 |
| Overweight or obese and Hyperuricemia (n/%) | 77 (21.33) | 31 (19.14) | 46 (23.12) | 0.359 |
| Table Notes：hypertension (55.68%), overweight or obesity (43.77%), and hyperuricemia (40.60%) were top 3 chronic diseases. | | | | |

Table S2 Logistic regression analysis of influencing factors of Hypertension

|  | | Univariable/adjusted models* | | | Original MV model* | | | Final MV model* | | |  |
| --- | --- | --- | --- | --- | --- | --- | --- | --- | --- | --- | --- |
| Predictor | Category | OR (SE) | 95% CI | p | OR (SE) | 95% CI | p | OR (SE) | 95% CI | P | |
| Overweight or obese | Yes | 1.53 (0.336) | 0.99 ,2.35 | 0.053 | 0.83(0.24) | 0.47,1.45 | 0.511 |  |  |  | |
| WC | cm | 1.05 (0.014) | 1.02 ,1.08 | <0.001 | 1.05 (0.02) | 1.02,1.09 | 0.004 | 1.05（0.01） | 1.02,1.07 | 0.001 | |
| Smoking | Yes | 0.52 (0.17) | 0.26 ,1.02 | 0.056 | 0.49 (0.17) | 0.24,0.98 | 0.044 |  |  |  | |
| Alcohol (reference=none) | None | 1 |  |  |  |  |  |  |  |  | |
|  | Occasional | 1.40 (0.53) | 0.67,2.92 | 0.374 |  |  |  |  |  |  | |
|  | Excessive | 0.99 (0.48) | 0.38,2.55 | 0.984 |  |  |  |  |  |  | |
| Physical activity behaviors (reference=none) | None | 1 |  |  |  |  |  |  |  |  | |
|  | Low level | 1.23(0.43) | 0.62,2.45 | 0.558 |  |  |  |  |  |  | |
|  | Moderate level | 1.25 (0.34) | 0.73,2.15 | 0.417 |  |  |  |  |  |  | |
|  | Vigorous level | 1.94 (1.27) | 0.54,6.97 | 0.308 |  |  |  |  |  |  | |
| Hypertension (UA) | Yes | 1.77(0.39) | 1.14,2.72 | 0.010 | 1.56(0.38) | 0.98,2.50 | 0.062 | 1.62（0.37） | 1.03,2.54 | 0.036 | |
| Hyperglycemia (FBG) | Yes | 1.48 (0.43) | 0.83,2.62 | 0.184 | 1.51(0.45) | 0.90,2.90 | 0.174 |  |  |  | |
| Hypercholesterolemia (TC) | Yes | 1.71 (0.51) | 0.96,3.05 | 0.069 | 1.61（0.48） | 0.90,2.90 | 0.111 |  |  |  | |
| Hypercholesterolemia (TG) | Yes | 0.99(0.31) | 0.54,1.784 | 0.980 |  |  |  |  |  |  | |
| Dyslipidemia (LDL) | Yes | 1.43 (0.48) | 0.74,2.77 | 0.286 |  |  |  |  |  |  | |
| Dyslipidemia (HDL) | Yes | 1.61(0.69) | 0.70,3.72 | 0.264 |  |  |  |  |  |  | |
| Fatty livers | Yes | 1.55 (0.36) | 0.98 ,2.44 | 0.058 | 1.02(0.27) | 0.61,1.73 | 0.932 |  |  |  | |
| Left ventricular hypertrophy | Yes | 3.04 (2.46) | 0.62 ,14.81 | 0.168 | 2.84 (2.06) | 0.69,11.77 | 0.150 |  |  |  | |
| Table Notes：*= all models adjusted by age and gender; UA= uric acid; FBG=fasting blood suger; TC= total cholesterol; TG= triglycerides; LDL= low-density lipoprotein; HDL= high-density lipoprotein. | | | | | | | | | | | |

Table S3 Logistic regression analysis of influencing factors of Hyperuricemia

|  | | Univariable/adjusted models* | | | Original MV model* | | | Final MV model * | | |
| --- | --- | --- | --- | --- | --- | --- | --- | --- | --- | --- |
| Predictor | Category | OR (SE) | 95% CI | p | OR (SE) | 95% CI | p | OR (SE) | 95% CI | P |
| Overweight or obese | Yes | 1.92(0.43) | 1.24,2.96 | 0.003 | 1.39(0.42) | 0.77,2.52 | 0.276 |  |  |  |
| WC | cm | 1.05(0.01) | 1.02,1.08 | <0.001 | 1.02(0.02) | 0.99,1.06 | 0.181 | 1.04(0.01) | 1.01,1.07 | 0.006 |
| Smoking | Yes | 1.78(0.61) | 0.91,3.50 | 0.094 | 2.53 (1.00) | 1.17, 5.48 | 0.018 | 2.40(0.92) | 1.13,5.07 | 0.022 |
| Alcohol | None | 1 |  |  |  |  |  |  |  |  |
|  | Occasional | 1.12 (0.41) | 0.55,2.30 | 0.753 |  |  |  |  |  |  |
|  | Excessive | 0.18 (0.14) | 0.04,0.81 | 0.025 |  |  |  |  |  |  |
| Physical activity behaviors | None | 1 |  |  |  |  |  |  |  |  |
|  | Low level | 0.81 (0.28) | 0.41,1.61 | 0.550 |  |  |  |  |  |  |
|  | Moderate level | 0.68(0.19) | 0.39,1.16 | 0.156 |  |  |  |  |  |  |
|  | Vigorous level | 0.56 (0.37) | 0.16,2.02 | 0.380 |  |  |  |  |  |  |
| Hypertension (SBP) | Yes | 1.77(0.39) | 1.14,2.72 | 0.010 | 1.62(0.40) | 1.00,2.64 | 0.050 | 1.66(0.41) | 1.03,2.68 | 0.039 |
| Hyperglycemia (FBG) | Yes | 0.60(0.18) | 0.33,1.08 | 0.089 | 0.40(0.13) | 0.21,0.77 | 0.006 | 0.40(0.13) | 0.21,0.77 | 0.006 |
| Hypercholesterolemia (TC) | Yes | 2.15(0.61) | 1.23,3.76 | 0.007 | 2.33(1.06) | 0.95,5.70 | 0.064 | 2.21(0.65) | 1.24,3.94 | 0.007 |
| Dyslipidemia (TG) | Yes | 3.22(1.04) | 1.71,6.09 | <0.001 | 2.48(1.04) | 1.09,5.65 | 0.031 | 2.79(1.09) | 1.29,6.01 | 0.009 |
| Dyslipidemia (LDL) | Yes | 2.12(0.69) | 1.12,4.00 | 0.020 | 1.00(0.52) | 0.36,2.76 | 0.997 |  |  |  |
| Dyslipidemia (HDL) | Yes | 4.87(2.25) | 1.97,12.03 | 0.001 | 4.06(2.39) | 1.28,12.90 | 0.018 | 4.12(2.44) | 1.29,13.15 | 0.017 |
| Fatty livers | Yes | 2.05(0.47) | 1.31,3.21 | 0.002 | 1.25(0.36) | 0.72,2.20 | 0.429 |  |  |  |
| Left ventricular hypertrophy | Yes | 3.11(2.19) | 0.78,12.38 | 0.107 | 2.53(1.73) | 0.66,9.69 | 0.174 |  |  |  |
| Table Notes：*= all models adjusted by age and gender; UA= uric acid; FBG=fasting blood suger; TC= total cholesterol; TG= triglycerides; LDL= low-density lipoprotein; HDL= high-density lipoprotein. | | | | | | | | | | |

Table S4 Logistic regression analysis of influencing factors of Hyperglycemia

|  | | Univariable/adjusted models* | | | Original MV model* | | | Final MV model* | | |
| --- | --- | --- | --- | --- | --- | --- | --- | --- | --- | --- |
| Predictor | Category | OR (SE) | 95% CI | p | OR (SE) | 95% CI | p | OR (SE) | 95% CI | P |
| Overweight or obese | Yes | 0.93 (0.27) | 0.53,1.64 | 0.797 |  |  |  |  |  |  |
| WC | cm | 1.01 (0.02) | 0.98,1.04 | 0.479 |  |  |  |  |  |  |
| Smoking | Yes | 1.04 (0.46) | 0.43,2.48 | 0.937 |  |  |  |  |  |  |
| Alcohol | None | 1 |  |  |  |  |  |  |  |  |
|  | Occasional | 1.50 (0.65) | 0.64,3.52 | 0.350 |  |  |  |  |  |  |
|  | Excessive | 1.09 (0.72) | 0.30,3.99 | 0.891 |  |  |  |  |  |  |
| Physical activity behaviors | None | 1 |  |  |  |  |  |  |  |  |
|  | Low level | 1.78 (0.82) | 0.72,4.39 | 0.212 |  |  |  |  |  |  |
|  | Moderate level | 1.15 (0.45) | 0.53,2.49 | 0.714 |  |  |  |  |  |  |
|  | Vigorous level | 1.22 (1.02) | 0.24,6.27 | 0.815 |  |  |  |  |  |  |
| Hypertension (SBP) | Yes | 1.48 (0.43) | 0.83,2.62 | 0.180 | 1.63(0.51) | 0.89,3.00 | 0.114 |  |  |  |
| Hyperuricemia (UA) | Yes | 0.60 (0.18) | 0.33,1.08 | 0.089 | 0.41(0.13) | 0.22,0.77 | 0.006 | 0.47(0.15) | 0.25,0.88 | 0.019 |
| Hypercholesterolemia (TC) | Yes | 1.08(0.40) | 0.52,2.25 | 0.831 |  |  |  |  |  |  |
| Dyslipidemia (TG) | Yes | 2.67(0.94) | 1.33,5.32 | 0.005 | 2.86 (1.14) | 1.31,6.25 | 0.009 | 3.38 (1.25) | 1.63,6.99 | 0.001 |
| Dyslipidemia (LDL) | Yes | 1.08(0.46) | 0.47,2.50 | 0.862 |  |  |  |  |  |  |
| Dyslipidemia (HDL) | Yes | 1.75 (0.83) | 0.70,4.42 | 0.232 |  |  |  |  |  |  |
| Fatty livers | Yes | 1.92 (0.55) | 1.10,3.38 | 0.023 | 1.66(0.51) | 0.91,3.02 | 0.099 |  |  |  |
| Left ventricular hypertrophy | Yes | 1.05 (0.91) | 0.19,5.75 | 0.954 |  |  |  |  |  |  |
| Table Notes：*= all models adjusted by age and gender; UA= uric acid; FBG=fasting blood suger; TC= total cholesterol; TG= triglycerides; LDL= low-density lipoprotein; HDL= high-density lipoprotein. | | | | | | | | | | |

Table S5 Logistic regression analysis of influencing factors of Hypercholesterolemia (TC)

|  | | Univariable/adjusted models* | | | Original MV model* | | | Final MV model* | | |
| --- | --- | --- | --- | --- | --- | --- | --- | --- | --- | --- |
| Predictor | Category | OR (SE) | 95% CI | p | OR (SE) | 95% CI | p | OR (SE) | 95% CI | P |
| Overweight or obese | Yes | 0.77(0.22) | 0.43,1.36 | 0.367 |  |  |  |  |  |  |
| WC | cm | 1.00(0.02) | 0.97,1.03 | 0.977 |  |  |  |  |  |  |
| Smoking | Yes | 0.82(0.40) | 0.32,2.12 | 0.684 |  |  |  |  |  |  |
| Alcohol | None | 1 |  |  |  |  |  |  |  |  |
|  | Occasional | 0.75 (0.40) | 0.26,2.12 | 0.583 |  |  |  |  |  |  |
|  | Excessive | 0.56 (0.44) | 0.12,2.66 | 0.463 |  |  |  |  |  |  |
| Physical activity behaviors | None | 1 |  |  |  |  |  |  |  |  |
|  | Low level | 0.92 (0.41) | 0.38,2.022 | 0.852 |  |  |  |  |  |  |
|  | Moderate level | 0.79 (0.28) | 0.40,1.57 | 0.508 |  |  |  |  |  |  |
|  | Vigorous level | 0.85 (0.70) | 0.17,4.31 | 0.842 |  |  |  |  |  |  |
| Hypertension (SBP) | Yes | 1.71(0.51) | 0.96,3.06 | 0.069 | 1.89(0.90) | 0.75,4.80 | 0.178 |  |  |  |
| Hyperuricemia (UA) | Yes | 2.15 (0.61) | 1.23,3.75 | 0.007 | 1.86(0.87) | 0.74,4,65 | 0.184 |  |  |  |
| Hyperglycemia (FBG) | Yes | 1.08(0.40) | 0.52,2.25 | 0.829 |  |  |  |  |  |  |
| Dyslipidemia (TG) | Yes | 2.16(0.77) | 1.07,4.35 | 0.031 | 0.98(0.69) | 0.24,3.91 | 0.974 |  |  |  |
| Dyslipidemia (LDL) | Yes | 2.78(1.95) | 7.02,11.02 | <0.001 | 2.70(1.92) | 0.67,10.89 | <0.001 | 2.78(1.95) | 0.70,11.02 | <0.001 |
| Dyslipidemia (HDL) | Yes | 0.38(0.28) | 0.09,1.66 | 0.196 | 0.52(0.46) | 0.09,2.97 | 0.465 |  |  |  |
| Fatty livers | Yes | 0.97(0.29) | 0.54,1.74 | 0.916 |  |  |  |  |  |  |
| Left ventricular hypertrophy | Yes | 0.49(0.57) | 0.05,4.74 | 0.539 |  |  |  |  |  |  |
| Table Notes：*= all models adjusted by age and gender; UA= uric acid; FBG=fasting blood suger; TC= total cholesterol; TG= triglycerides; LDL= low-density lipoprotein; HDL= high-density lipoprotein. | | | | | | | | | | |

Table S6 Logistic regression analysis of influencing factors of Hypercholesterolemia (TG)

|  | | Univariable/adjusted models* | | | Original MV model* | | | Final MV model* | | |
| --- | --- | --- | --- | --- | --- | --- | --- | --- | --- | --- |
| Predictor | Category | OR (SE) | 95% CI | p | OR (SE) | 95% CI | p | OR (SE) | 95% CI | P |
| Overweight or obese | Yes | 1.52(0.46) | 0.83,2.76 | 0.175 | 0.55(0.26) | 0.22,1.38 | 0.204 |  |  |  |
| WC | cm | 1.05(0.02) | 1.02,1.09 | 0.002 | 1.02(0.03) | 0.97,1.07 | 0.407 |  |  |  |
| Smoking | Yes | 0.82(0.46) | 0.27,2.44 | 0.718 |  |  |  |  |  |  |
| Alcohol | None | 1 |  |  |  |  |  |  |  |  |
|  | Occasional | 0.58 (0.39) | 0.16,2.16 | 0.419 |  |  |  |  |  |  |
|  | Excessive | 0.75 (0.60) | 0.16,3.56 | 0.720 |  |  |  |  |  |  |
| Physical activity behaviors | None | 1 |  |  |  |  |  |  |  |  |
|  | Low level | 2.27 (1.14) | 0.85,6.07 | 0.103 |  |  |  |  |  |  |
|  | Moderate level | 1.11(0.49) | 0.47,2.64 | 0.814 |  |  |  |  |  |  |
|  | Vigorous level | 1.71 (1.52) | 0.30,9.73 | 0.544 |  |  |  |  |  |  |
| Hypertension (SBP) | Yes | 0.99(0.31) | 0.54,1.84 | 0.986 |  |  |  |  |  |  |
| Hyperuricemia (UA) | Yes | 3.22(1.04) | 1.71,6.08 | <0.001 | 1.85(0.71) | 0.87,3.94 | 0.108 |  |  |  |
| Hyperglycemia (FBG) | Yes | 2.66(0.93) | 1.33,5.29 | 0.006 | 2.02(0.90) | 0.85,4.83 | 0.111 |  |  |  |
| Dyslipidemia (TC) | Yes | 2.16(0.77) | 1.07,4.35 | 0.031 | 1.62(1.01) | 0.49,5.46 | 0.419 |  |  |  |
| Dyslipidemia (LDL) | Yes | 3.00(1.16) | 1.41,6.40 | 0.004 | 2.12(1.37) | 0.60,7.54 | 0.245 | 3.91(1.61) | 1.77,8.77 | 0.001 |
| Dyslipidemia (HDL) | Yes | 7.48(3.23) | 3.21,17.44 | <0.001 | 5.47(3.06) | 1.82,16.37 | 0.002 | 6.19(3.13) | 2.30,16.68 | <0.001 |
| Fatty livers | Yes | 8.07(2.90) | 3.99,16.32 | <0.001 | 6.47(2.67) | 2.88,14.52 | <0.001 | 6.84(2.53) | 3.30,14.14 | <0.001 |
| Left ventricular hypertrophy | Yes | 2.99(2.23) | 0.69,12.88 | 0.142 | 2.88(2.66) | 0.47,17.64 | 0.252 |  |  |  |
| Table Notes：*= all models adjusted by age and gender; UA= uric acid; FBG=fasting blood suger; TC= total cholesterol; TG= triglycerides; LDL= low-density lipoprotein; HDL= high-density lipoprotein. | | | | | | | | | | |

Table S7 Logistic regression analysis of influencing factors of Dyslipidemia (LDL)

|  | | Univariable/adjusted models* | | | Original MV model* | | | Final MV model* | | |
| --- | --- | --- | --- | --- | --- | --- | --- | --- | --- | --- |
| Predictor | Category | OR (SE) | 95% CI | p | OR (SE) | 95% CI | p | OR (SE) | 95% CI | P |
| Overweight or obese | Yes | 0.91(0.30) | 0.48,1.72 | 0.766 |  |  |  |  |  |  |
| WC | cm | 1.03(0.02) | 0.99,1.07 | 0.110 | 1.08(0.03) | 1.02,1.15 | 0.008 | 1.09(0.03) | 1.03,1.16 | 0.005 |
| Smoking | Yes | 0.79(0.40) | 0.29,2.15 | 0.645 |  |  |  |  |  |  |
| Alcohol | None | 1 |  |  |  |  |  |  |  |  |
|  | Occasional | 0.69 (0.41) | 0.21,2.20 | 0.525 |  |  |  |  |  |  |
|  | Excessive | 0.79 (0.40) | 0.29,2.15 | 0.652 |  |  |  |  |  |  |
| Physical activity behaviors | None | 1 |  |  |  |  |  |  |  |  |
|  | Low level | 1.03(0.55) | 0.36,2.91 | 0.958 |  |  |  |  |  |  |
|  | Moderate level | 0.95 (0.39) | 0.42,2.12 | 0.900 |  |  |  |  |  |  |
|  | Vigorous level | 0.86 (0.57) | 0.23,3.16 | 0.827 |  |  |  |  |  |  |
| Hypertension (SBP) | Yes | 1.43(0.48) | 0.74,2.77 | 0.282 |  |  |  |  |  |  |
| Hyperglycemia (FBG) | Yes | 1.07(0.46) | 0.46,2.49 | 0.869 |  |  |  |  |  |  |
| Hyperuricemia (UA) | Yes | 2.11(0.69) | 1.12,3.99 | 0.020 | 0.87(0.49) | 0.29,2.61 | 0.799 | 1.99(0.66) | 1.04, 3.83 | 0.038 |
| Dyslipidemia (TC) | Yes | 2.83(20.01) | 7.09,11.30 | <0.001 | 4.00(3.18) | 0.84,19.01 | <0.001 | 3.89(2.98) | 0.86,17.51 | <0.001 |
| Dyslipidemia (TG) | Yes | 2.99(1.15) | 1.40,6.37 | 0.005 | 3.40(3.17) | 0.55,21.19 | 0.190 |  |  |  |
| Dyslipidemia (HDL) | Yes | 0.24(0.25) | 0.03,1.85 | 0.172 | 0.06(0.07) | 0.01,0.52 | 0.011 |  |  |  |
| Fatty livers | Yes | 1.43(0.48) | 0.74,2.76 | 0.285 | 1.14(0.78) | 0.29,4.36 | 0.848 |  |  |  |
| Left ventricular hypertrophy | Yes | 0.76(0.82) | 0.92,6.32 | 0.801 |  |  |  |  |  |  |
| Table Notes：*= all models adjusted by age and gender; UA= uric acid; FBG=fasting blood suger; TC= total cholesterol; TG= triglycerides; LDL= low-density lipoprotein; HDL= high-density lipoprotein. | | | | | | | | | | |

Table S8 Logistic regression analysis of influencing factors of Dyslipidemia (HDL)

|  | | Univariable/adjusted models* | | | Original MV model* | | | Final MV model* | | |
| --- | --- | --- | --- | --- | --- | --- | --- | --- | --- | --- |
| Predictor | Category | OR (SE) | 95% CI | p | OR (SE) | 95% CI | p | OR (SE) | 95% CI | P |
| Overweight or obese | Yes | 2.57(1.09) | 1.12,5.89 | 0.026 | 1.28(0.64) | 0.48,3.42 | 0.629 |  |  |  |
| WC | cm | 1.07(0.02) | 1.02,1.11 | 0.003 | 1.03(0.03) | 0.97,1.10 | 0.338 |  |  |  |
| Smoking | Yes | 0.99(0.56) | 0.32,3.02 | 0.980 |  |  |  |  |  |  |
| Alcohol | None | 1 |  |  |  |  |  |  |  |  |
|  | Occasional | 0.23(0.24) | 0.03,1.77 | 0.157 |  |  |  |  |  |  |
|  | Excessive | 0.45(0.49) | 0.05,3.77 | 0.465 |  |  |  |  |  |  |
| Physical activity behaviors | None | 1 |  |  |  |  |  |  |  |  |
|  | Low level | 0.74(0.50) | 0.19,2.82 | 0.656 |  |  |  |  |  |  |
|  | Moderate level | 0.76 (0.38) | 0.28(2.05) | 0.585 |  |  |  |  |  |  |
|  | Vigorous level | 2.00(1.70) | 0.38(10.59) | 0.413 |  |  |  |  |  |  |
| Hypertension (SBP) | Yes | 1.62(0.69) | 0.70,3.73 | 0.257 |  |  |  |  |  |  |
| Hyperglycemia (FBG) | Yes | 1.74(0.82) | 0.69,4.40 | 0.241 |  |  |  |  |  |  |
| Hyperuricemia (UA) | Yes | 4.85(2.24) | 1.96,11.99 | 0.001 | 4.50(2.28) | 1.66,12.17 | 0.003 | 4.44(2.26) | 1.64,12.05 | 0.003 |
| Dyslipidemia (TC) | Yes | 0.38(0.29) | 0.09,1.66 | 0.197 | 0.69（0.78） | 0.08,6.24 | 0.744 |  |  |  |
| Dyslipidemia (TG) | Yes | 7.42(3.21) | 3.18,17.31 | <0.001 | 5.35(3.00) | 1.78,16.05 | 0.003 | 5.32(3.00) | 1.76,16.10 | 0.003 |
| Dyslipidemia (LDL) | Yes | 0.24(0.25) | 0.03,1.86 | 0.172 | 0.11(0.16) | 0.006,2.00 | 0.136 |  |  |  |
| Fatty livers | Yes | 4.74(2.04) | 2.04,11.03 | <0.001 | 2.13(1.13) | 0.75,6.04 | 0.156 | 2.86(1.52) | 1.01,8.10 | 0.047 |
| Table Notes：*= all models adjusted by age and gender; UA= uric acid; FBG=fasting blood suger; TC= total cholesterol; TG= triglycerides; LDL= low-density lipoprotein; HDL= high-density lipoprotein. | | | | | | | | | | |

Table S9 Logistic regression analysis of influencing factors of Fatty liver disease

|  | | Univariable/adjusted models* | | | Original MV model* | | | Final MV model* | | |
| --- | --- | --- | --- | --- | --- | --- | --- | --- | --- | --- |
| Predictor | Category | OR (SE) | 95% CI | p | OR (SE) | 95% CI | p | OR (SE) | 95% CI | P |
| Overweight or obese | Yes | 5.31(1.30) | 3.29,8.57 | <0.001 | 3.11(1.02) | 1.63,5.91 | 0.001 | 3.22(1.03) | 1.72,6.03 | <0.001 |
| WC | cm | 1.12(0.02) | 1.08,1.16 | <0.001 | 1.07(0.02) | 1.02,1.12 | 0.003 | 1.07(0.02) | 1.03,1.12 | 0.001 |
| Smoking | Yes | 0.62(0.23) | 0.29,1.29 | 0.197 | 0.75(0.33) | 0.32,179 | 0.521 |  |  |  |
| Alcohol |  |  |  |  |  |  |  |  |  |  |
|  | Occasional | 1.07 (0.40) | 0.51,2.22 | 0.865 |  |  |  |  |  |  |
|  | Excessive | 0.57 (0.33) | 0.19,1.74 | 0.325 |  |  |  |  |  |  |
| Physical activity behaviors |  |  |  |  |  |  |  |  |  |  |
|  | Low level | 1.29(0.49) | 0.62,2.71 | 0.496 |  |  |  |  |  |  |
|  | Moderate level | 1.08 (0.33) | 0.60,1.96 | 0.788 |  |  |  |  |  |  |
|  | Vigorous level | 1.49 (0.97) | 0.42,5.36 | 0.538 |  |  |  |  |  |  |
| Hypertension (SBP) | Yes | 1.56(0.36) | 0.99,2.46 | 0.057 | 1.13(0.32) | 0.65,1.96 | 0.668 |  |  |  |
| Hyperglycemia (FBG) | Yes | 1.92(0.55) | 1.09,3.36 | 0.023 | 1.71(0.59) | 0.87,3.37 | 0.119 |  |  |  |
| Hyperuricemia (UA) | Yes | 2.05(0.47) | 1.31,3.22 | 0.002 | 1.19(0.35) | 0.67,2.13 | 0.552 |  |  |  |
| Dyslipidemia (TC) | Yes | 0.97(0.29) | 0.54,1.74 | 0.931 |  |  |  |  |  |  |
| Dyslipidemia (TG) | Yes | 8.05(2.88) | 4.00,16.22 | <0.001 | 6.70(2.81) | 2.94,15.26 | <0.001 | 8.58(3.39) | 3.95,18.61 | <0.001 |
| Dyslipidemia (LDL) | Yes | 1.43(0.48) | 0.74,2.75 | 0.284 |  |  |  |  |  |  |
| Dyslipidemia (HDL) | Yes | 4.69(2.01) | 2.03,10.84 | <0.001 | 2.16(1.19) | 0.73,6.36 | 0.164 |  |  |  |
| Left ventricular hypertrophy | Yes | 2.59(1.68) | 0.72,9.26 | 0.144 | 1.69(1.18) | 0.43,6.67 | 0.451 |  |  |  |
| Table Notes：*= all models adjusted by age and gender; UA= uric acid; FBG=fasting blood suger; TC= total cholesterol; TG= triglycerides; LDL= low-density lipoprotein; HDL= high-density lipoprotein. | | | | | | | | | | |

Table S10 STROBE Statement—Checklist of items that should be included in reports of cross-sectional studies

|  | Item No | Recommendation | Pages |
| --- | --- | --- | --- |
| **Title and abstract** | 1 | (*a*) Indicate the study’s design with a commonly used term in the title or the abstract | 1 |
|  |  | (*b*) Provide in the abstract an informative and balanced summary of what was done and what was found | 3 |
| Introduction | | |  |
| Background/rationale | 2 | Explain the scientific background and rationale for the investigation being reported | 5 |
| Objectives | 3 | State specific objectives, including any prespecified hypotheses | 8 |
| Methods | | |  |
| Study design | 4 | Present key elements of study design early in the paper | 9 |
| Setting | 5 | Describe the setting, locations, and relevant dates, including periods of recruitment, exposure, follow-up, and data collection | 10 |
| Participants | 6 | (*a*) Give the eligibility criteria, and the sources and methods of selection of participants | 9 |
| Variables | 7 | Clearly define all outcomes, exposures, predictors, potential confounders, and effect modifiers. Give diagnostic criteria, if applicable | 10 |
| Data sources/ measurement | 8* | For each variable of interest, give sources of data and details of methods of assessment (measurement). Describe comparability of assessment methods if there is more than one group | *8* |
| Bias | 9 | Describe any efforts to address potential sources of bias | 9 |
| Study size | 10 | Explain how the study size was arrived at | 10 |
| Quantitative variables | 11 | Explain how quantitative variables were handled in the analyses. If applicable, describe which groupings were chosen and why | 10 |
| Statistical methods | 12 | (*a*) Describe all statistical methods, including those used to control for confounding | 13 |
|  |  | (*b*) Describe any methods used to examine subgroups and interactions | 13 |
|  |  | (*c*) Explain how missing data were addressed | 13 |
|  |  | (*d*) If applicable, describe analytical methods taking account of sampling strategy | 13 |
|  |  | (*e*) Describe any sensitivity analyses | 13 |
| Results | | |  |
| Participants | 13* | (a) Report numbers of individuals at each stage of study—eg numbers potentially eligible, examined for eligibility, confirmed eligible, included in the study, completing follow-up, and analysed | 15 |
|  |  | (b) Give reasons for non-participation at each stage | NG |
|  |  | (c) Consider use of a flow diagram | NG |
| Descriptive data | 14* | (a) Give characteristics of study participants (eg demographic, clinical, social) and information on exposures and potential confounders | 15 |
|  |  | (b) Indicate number of participants with missing data for each variable of interest | 15 |
| Outcome data | 15* | Report numbers of outcome events or summary measures | 15 |
| Main results | 16 | (*a*) Give unadjusted estimates and, if applicable, confounder-adjusted estimates and their precision (eg, 95% confidence interval). Make clear which confounders were adjusted for and why they were included | 16 |
|  |  | (*b*) Report category boundaries when continuous variables were categorized | 15 |
|  |  | (*c*) If relevant, consider translating estimates of relative risk into absolute risk for a meaningful time period | 15 |
| Other analyses | 17 | Report other analyses done—eg analyses of subgroups and interactions, and sensitivity analyses | 16 |
| Discussion | | |  |
| Key results | 18 | Summarise key results with reference to study objectives | 17 |
| Limitations | 19 | Discuss limitations of the study, taking into account sources of potential bias or imprecision. Discuss both direction and magnitude of any potential bias | 22 |
| Interpretation | 20 | Give a cautious overall interpretation of results considering objectives, limitations, multiplicity of analyses, results from similar studies, and other relevant evidence | 17 |
| Generalisability | 21 | Discuss the generalisability (external validity) of the study results | 23 |
| Other information | | |  |
| Funding | 22 | Give the source of funding and the role of the funders for the present study and, if applicable, for the original study on which the present article is based | 23 |
| *Give information separately for exposed and unexposed groups.  **Note:** An Explanation and Elaboration article discusses each checklist item and gives methodological background and published examples of transparent reporting. The STROBE checklist is best used in conjunction with this article (freely available on the Web sites of PLoS Medicine at http://www.plosmedicine.org/, Annals of Internal Medicine at http://www.annals.org/, and Epidemiology at http://www.epidem.com/). Information on the STROBE Initiative is available at www.strobe-statement.org. | | | |
